# Thrombotic microangiopathy in juvenile dermatomyositis

**DOI:** 10.1186/1546-0096-9-S1-P49

**Published:** 2011-09-14

**Authors:** A Frolenko, N Bervina, M Kagan

**Affiliations:** 1 Department of Pediatric Rheumatology, Orenburg Regional Children’s Hospital, Orenburg, Russia; 2 Department of Pediatric Nephrology, Orenburg Regional Children’s Hospital, Orenburg, Russia

## Objectives and study

A 4- year-old boy was admitted to our hospital because of progressive proximal muscular weakness of 4 weeks duration, heliotrope rash, erythematous skin lesions in the trunk and limbs and febrile fever. The muscle enzymes were elevated: creatine kinase 7,056 IU/L (0–170), aldolase 40.3 U/L (0–8), aspartate transaminase 601 U/L (10–37), alanine transaminase 289 U/L (10–37). The laboratory test revealed a hemoglobin level of 86 g/dL, a platelet count of 100,000/mm3, an erythrocyte sedimentation rate of 30 mm/h . A peripheral blood smear revealed schistocytes (2%). Urine analysis revealed large number of red blood cells and proteinuria 1.06 grams / liter. The level of total serum bilirubin was 37 µmol / l (normal up to 17 µmol / l). Serum creatinine was 200 µmol/l. Direct and indirect Coombs' reaction was negative. Prothrombin and thrombin time and D-dimers were within normal limits.The serologic tests for the antinuclear antibody, antineutrophil cytoplasmic antibody, antibodies to DNA, cardiolipin antibodies, antibodies to β2-glycoproteins were negative. Lupus anticoagulant was not detected. The electromyographic findings were consistent with the inflammatory myopathy. A renal biopsy contained 43 glomeruli and was evaluated by light and immunofluorescence microscopies. Diffuse fibrin-platelet thrombi were revealed in glomerular capillaries. Small arteries was narrowed by the swelling of the endothelium. The expression of the fibrin + +. There were no immune deposits at any location.

## Methods

The patient received three pulses of methylprednisolone 30mg /kg daily, followed by oral prednisolone 60 mg/day. Alternate-day plasmapheresis with fresh frozen plasma for 1 week and intravenous cyclophosphamide treatment (750 mg/m^2^/month) were started.

## Results

The treatment resulted in slow improvement in his muscle weakness within 4 weeks along with markedly decreased muscle enzymes and normalization of renal function. Prednisolone was changed to 1 mg/day of oral prednisolone, which was later decreased to 0.5 mg/day followed by transition to long-term oral methotrexate 10 mg / m2/week (after 6 monthly pulses of cyclophosphamide).

## Conclusions

The described clinical case demonstrates the varied nature of renal pathology at juvenile dermatomyositis. The kidney damage appeared to have been very severe. But under the well-timed and adequate therapy it underwent to a back development in a great degree.

